# Learning implicit sentiments in Alzheimer's disease recognition with contextual attention features

**DOI:** 10.3389/fnagi.2023.1122799

**Published:** 2023-05-17

**Authors:** Ning Liu, Zhenming Yuan, Yan Chen, Chuan Liu, Lingxing Wang

**Affiliations:** ^1^School of Science/School of Big Data Science, Zhejiang University of Science and Technology, Hangzhou, China; ^2^School of Information Science and Technology, Hangzhou Normal University, Hangzhou, Zhejiang, China; ^3^International Unresponsive Wakefulness Syndrome and Consciousness Science Institute, Hangzhou Normal University, Hangzhou, China; ^4^School of Mathematics and Computer Science, Quanzhou Normal University, Quanzhou, Fujian, China; ^5^Department of Neurology, Second Affiliated Hospital of Fujian Medical University, Quanzhou, Fujian, China

**Keywords:** Alzheimer's disease, attention, deep learning, feature extraction, machine learning

## Abstract

**Background:**

Alzheimer's disease (AD) is difficult to diagnose on the basis of language because of the implicit emotion of transcripts, which is defined as a supervised fuzzy implicit emotion classification at the document level. Recent neural network-based approaches have not paid attention to the implicit sentiments entailed in AD transcripts.

**Method:**

A two-level attention mechanism is proposed to detect deep semantic information toward words and sentences, which enables it to attend to more words and fewer sentences differentially when constructing document representation. Specifically, a document vector was built by progressively aggregating important words into sentence vectors and important sentences into document vectors.

**Results:**

Experimental results showed that our method achieved the best accuracy of 91.6% on annotated public Pitt corpora, which validates its effectiveness in learning implicit sentiment representation for our model.

**Conclusion:**

The proposed model can qualitatively select informative words and sentences using attention layers, and this method also provides good inspiration for AD diagnosis based on implicit sentiment transcripts.

## 1. Introduction

Alzheimer's disease (AD) is a progressive degeneration of the brain and is irreversible (Mattson, 2004), and early diagnosis and intervention are essential as there is currently no optimal method to cure AD. A previous study (Mueller et al., 2018) showed that the first sign of the disease is the deterioration of language; therefore, early diagnosis based on language has gradually become a research hotspot. With the development of artificial intelligence (AI), natural language processing (NLP), and machine learning technology, diagnosing AD through these new technologies is possible, and AI technology based on language may be used as a preliminary diagnosis tool for people with cognitive impairment, which is indeed a text classification problem in the NLP area.

**Figure 1:**
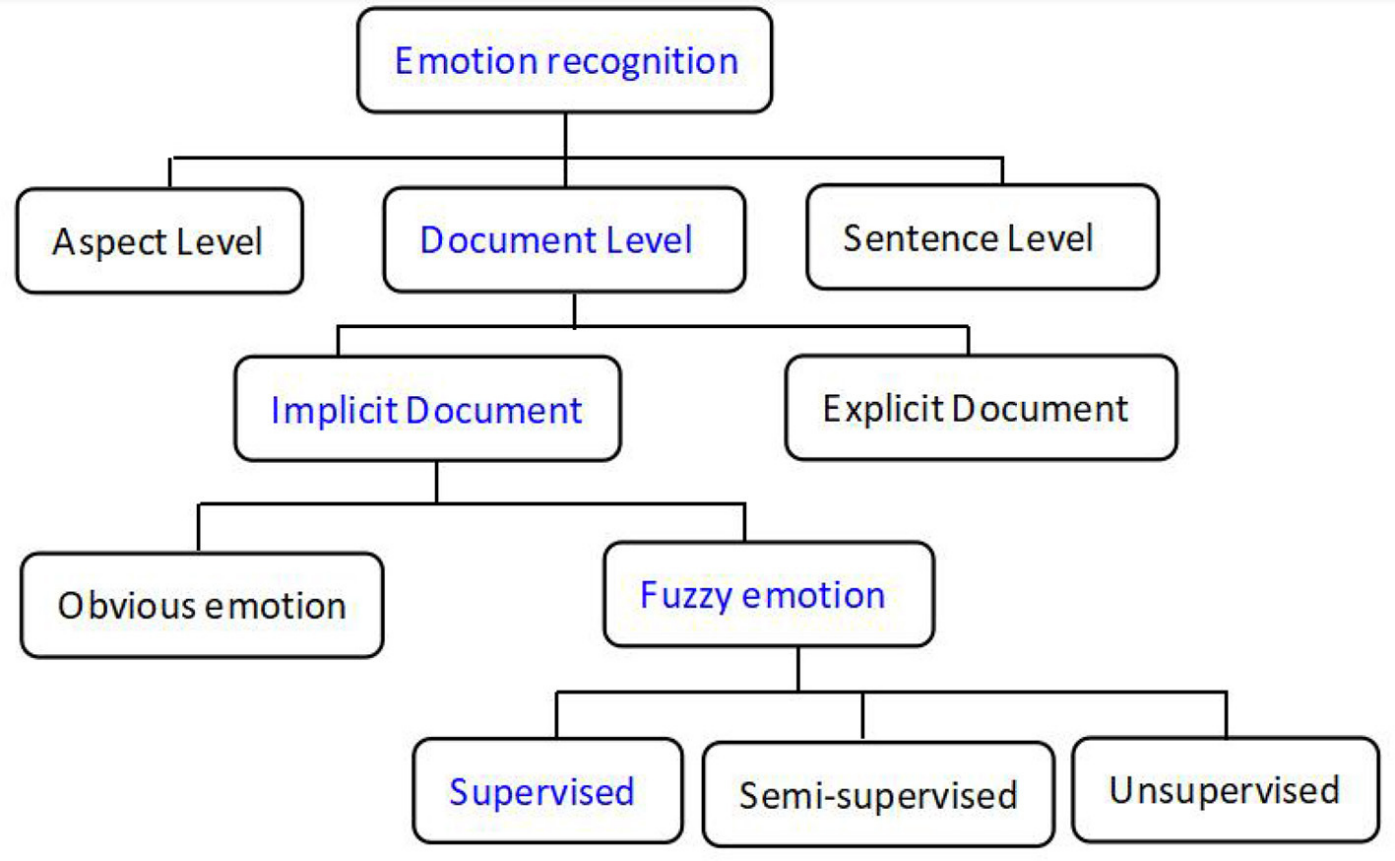
Classification of emotional recognition (blue is the character of the transcripts in this study).

Emotion recognition (text classification) can be classified into three levels according to previous studies (Medhat et al., 2014; Yadollahi et al., 2017), namely, the aspect, sentence, and document levels (Xu et al., 2015; Yadollahi et al., 2017), as shown in Figure 1. Meanwhile, texts at the document level can be classified as explicit or implicit emotions. Explicit sentiment refers to the obvious emotional words used to express sentiment polarity, and the classification model can extract these key emotional words and provide a large weight to perform the classification task accurately. Unlike explicit expressions, implicit sentiment analysis indicates that the sentences have no obvious emotional words but can still convey a clear sentiment polarity in the context (Russo et al., 2015). The model cannot extract these important emotional words for text classification correctly, which may lead to worse classification performance.

Reviews of explicit and implicit sentiments are presented in Table 1. In explicit expression, words such as “lovely”, “beautiful”, “bad”, and “like” have an obvious feeling tendency that can be captured toward a particular aspect by the classification model. Implicit sentiments may express emotions that cannot be easily found, such as irony, anger, and depression. According to a previous study (Xu et al., 2015), approximately 30% of reviews contain implicit aspects of emotional classification. For example, the sentence “We cannot bite the dog anymore when bitten by a mad dog” obviously expresses a sense of irony and negativity. “Sales of your company in a year cannot match us for a month” also expresses a negative meaning that indicates a poor sale. “The waiter poured water over me and walked away” means poor service, and although it contains no opinion words, it can be clearly interpreted as negative. These sentences must extract deep semantic information to be correctly classified. However, the text in this study is clearly different from explicit and implicit expressions as it does not have any emotional words or tendencies. An example of our transcripts is presented below.

*The scene is in the in the kitchen. The mother is wiping dishes and the water is running on the floor, a child is trying to get a boy is trying to get cookies outta out a jar and he's about to tip over on a stool. The little girl is reacting to his falling, it seems to be summer out, the window is open*.

**Table 1:** Reviews containing explicit and implicit sentiments.

**Explicit expression**	What a **lovely** girl!
	It's a **beautiful** day. I **like** it.
	The service of this hotel is **bad**, I must complain.
**Implicit expression**	Yeah, we can't bite the dog anymore when bitten by a mad dog.
	Sales of your company in a year cannot match us for a month.
	The waiter poured water over me and walked away

Bold means obvious emotional words.

The text above is an example of our dataset that has no emotional words and only a description of a picture. The famous Boston Diagnostic Aphasia Examination (Chen et al., 2019) was used for AD diagnosis. However, our text is an implicit expression and cannot convey a clear sentiment polarity in the context. In addition, humans cannot even judge emotional polarity from the text. Thus, texts with these characteristics are called “fuzzy emotions”. Though an implicit expression in the text, humans can judge the emotional polarity of the text, which is called “obvious emotion” in the implicit document. Fuzzy emotional document classification includes unsupervised, supervised, and semi-supervised methods. In this study, transcripts from voice recordings for AD diagnosis were supervised by the fuzzy implicit emotion classification at the document level. Sentiment analysis classification is shown in Figure 1.

For the classification of implicit transcripts with a long document in this study, the text lacks emotional words and context-dependent features. Compared with the explicit classification task, it is more difficult to perform classification tasks for fuzzy implicit text because it lacks obvious emotional words and polarity, and a deep-learning model cannot extract effective features from the transcripts, although extracting the features of fuzzy implicit documents is essential for AD diagnosis. In this study, a classification model combining the attention mechanism of words and sentence levels was designed in view of the dependence of implicit expression in contextual content. Not all words and sentences in the text are equally relevant to the final classification, and previous deep learning models paid little attention to words and sentences with different levels of importance for the classification correctly. Specifically, the bidirectional gated recurrent unit (GRU) was used to obtain vectors from the transcript, and an attention mechanism based on word and sentence levels was used to extract deep semantic features for better representation. Experiments showed that the accuracy on public Pitt datasets with five-cross validation was 91.6%, which is a competitive performance compared with other similar studies.

## 2. Related work

### 2.1. Implicit sentiment classification

Many studies have mentioned the presence of implicit sentiments in text classification. For example, Toprak et al. (2010) and Russo et al. (2015) proposed implicit polarity (polar facts) and provided a corpus with an implicit sentiment. Choi and Wiebe (2014) proposed a +/- EffectWordNet lexicon to recognize implicit sentiment, assuming that sentiment analysis was related to states and events which had a positive or negative effect on the entity. Deng and Wiebe (2014) detected implicit sentiment via inference over explicit expressions and the so-called goodFor/badFor events. Memory networks (Tang et al., 2016; Chen et al., 2017; Wang et al., 2018), graph neural networks (Sun et al., 2019; Zhang et al., 2019; Wang et al., 2020), and pretrained knowledge (Xu et al., 2019; Rietzler et al., 2020; Dai et al., 2021) were all used to capture aspect-related information from the text. Meanwhile, some studies used the attention mechanism, which was first proposed by Bahdanau et al. (2014) for machine translation, to extract implicit sentiment. It usually has better performance as it can extract the importance of different parts in texts. For example, a study by He et al. (2018) used syntax information from a dependency tree to enhance the attention-based model. The studies by Toprak et al. (2010) and Zehra et al. (2021) used different attention mechanisms to identify aspect-related contexts. In the study by He et al. (2018), two methods were proposed to improve attention effectiveness. First, they introduced an attention model that incorporates syntactic contents into the attention mechanism. Second, they proposed a method for target representation that could better capture the semantic meaning of the opinion target. In a study by Tang et al. (2020), a dependency graph enhanced a dual-transformer network with a dual-transformer structure to support the reinforcement of graph-based representation learning. Ma et al. (2017) proposed an interactive attention network to learn the relationship between contexts and targets, which is mainly based on the concept that both contexts and targets should be treated specifically. Wang et al. (2016) proposed an attention-based long short-term memory (LSTM) network for aspect-level text classification and obtained state-of-the-art performance on SemEval 2014 datasets. However, these studies are all implicit classifications with obvious emotions, and to the best of our knowledge, there are no studies of fuzzy implicit emotion classification other than those in the AD diagnosis area.

### 2.2. AD diagnosis based on acoustic and its transcripts

There are three main methods to recognize AD and MCI from normal control (NC) in this area. The first method uses traditional machine learning methods in combination with manual feature extraction, which needs professional knowledge to extract effective features. Although the explanation of this method is better, the performance is just maybe passable. The second approach uses deep learning models to recognize AD and MCI, the performance of which is usually better than the first method. However, the interpretability is not better as deep learning is a “black” box and it is difficult to understand the meaning of the features extracted automatically. The third approach is a combination of the first two methods and may further improve the performance of deep learning. It highlights the important linguistic or phonetic features in participant language description tasks, which may have a significant guide for AD clinical diagnosis.

The first method uses manual conventional, phonetic, and linguistic feature extraction as key factors. For example, the study by Luz S. (2017), to the best of our knowledge, was the first to employ speech datasets exclusively for analysis without transcripts, extract low-level acoustic features, such as speech rate, vocalization events, and the number of utterances, use Bayesian classifiers to train on low speech datasets extracted from the recordings, and achieve 68% accuracy in classifying AD and elderly controls. Fraser et al. (2016) extracted 42 mel-frequency cepstral coefficient (MFCC) features (Chen et al., 2014) from Pitt datasets and is the first study to carry out an acoustic-prosodic analysis. Another study by Roark et al. (2011) employed automatic speech recognition (ASR) and natural language processing (NLP) to classify MCI and healthy participants; the extracted features included pause frequency and duration. Finally, the SVM classifier obtained the best AUC of 0.861 by combining linguistic features, automated speech, and cognitive test scores. Jarrold et al. (2014) extracted 41 features, including the mean and standard deviation of the duration of pauses, speech rate, and consonants and vowels. The datasets included nine AD patients, 13 semantic dementia patients, nine healthy controls, nine frontotemporal dementia patients, and eight progressive nonfluent aphasia patients. Zehra et al. (2021) extracted speech rate (Luz, 2013) and graph-based features by encoding patterns from Carolina Conversations Collection (Pope and Davis, 2011) and used the logistic regression classifier to obtain an accuracy of 85% when distinguishing AD from non-AD participants. Toth et al. (2018) found that a pause could not be detected reliably by human annotators, whereas using an ASR system improved the effectiveness. They analyzed the speech of 48 MCI and 38 healthy controls and extracted acoustic features such as the length of utterance, hesitation ratio, filled pauses, and speech tempo. Finally, ASR-extracted features in combination with a Random forest classifier manifested the best results (75% accuracy). For example, Antonsson et al. (2021) quantitatively measured the semantic ability, used the Support Vector Machine (SVM) classifier to recognize AD, and finally obtained the best area under the curve (AUC) of 0.93. Clarke et al. (2013) measured 286 linguistic features to train the SVM classifier, and the final accuracy obtained was 50–78% for MCI vs. HC, 59–90% for AD vs. HC, and 62–78% for AD+MCI vs. HC. Meanwhile, the study found that the speech task impacts the accuracy of AD detection more than the length of the sample. R'mani and James (2021) investigated the use of x-vector and i-vector methods (Snyder et al., 2018) that were linguistic features for tackling AD detection and phonetic features devised originally for speaker identification and yielded 85.4% accuracy in AD detection with Random Forests and SVM. Shamila et al. (2021) used the Carolinas Conversations Collection Classification Model (Pope and Davis, 2011), investigated conversational features such as pauses, dysfluencies, overlaps, and other elements for AD detection, and finally achieved the best accuracy of 90% in Alzheimer's Dementia Recognition through Spontaneous Speech (ADReSS) datasets. Zehra et al. (2021) developed acoustic and linguistic features by combining a regularized logistic regression classifier, achieving an accuracy of 85.4% on DementiaBank datasets.

Deep learning models for AD recognition by the second method include Convolutional Neural Networks (CNN), Recurrent Neural Networks (RNN), LSTM, and Transformer and BERT. For instance, in the study by Fritsch et al. (2019), the n-gram language model was enhanced by creating a neural network language model with LSTM and finally obtained an accuracy of 85.6%. A study by Chen et al. (2019) proposed a network based on the attention mechanism composed of GRU and CNN modules and finally obtained a state-of-the-art accuracy of 97% in distinguishing individuals with AD from NC. Balagopalan et al. (2021) used a pretrained BERT model to recognize AD from NC with ADReSS datasets and achieved an accuracy of 83.33%, thus outperforming the performance of acoustic and linguistic features manually. Guo et al. (2021) trained a BERT model on DementiaBank and ADReSS datasets with different sizes and demonstrated that more datasets can obtain a better performance than minor datasets relatively. Meghanani et al. (2021) compared two approaches for AD recognization—one method employed the fastText model and the other used the CNN model. The performance of the fastText model outperformed the CNN model and achieved the best accuracy of 83.3% in classification.

The third method can combine the advantage of the first two methods—deep learning models combined with acoustic features or linguistic features can manually improve the performance of the model further. For example, the champion of the Interspeech challenge in 2020 (Yuan et al., 2020), the world's premier conference on speech research, combined the Baidu ERNIE model and pause information with three different sizes (extracted with Penn Phonetics Lab Forced Aligner) and finally achieved the best accuracy of 89.6%. From this study, we can conclude that pause is an important and distinguishing feature of AD recognition. Pranav and Veeky (2021) employed a deep learning model in combination with the acoustic and linguistic features on ADReSS (78 AD vs. 78 HC) datasets and DementiaBank datasets, respectively. The performance of the model that combines linguistic features was better than the model that combines the acoustic features, with accuracies of 88% and 73%, respectively. This method, to the best of our knowledge, is the most promising research direction of the future.

## 3. Attention network

### 3.1. GRU-based sequence encoder

GRU is a variant structure of LSTM (Hochreiter and Schmidhuber, 1997), which can effectively solve the problem of gradient vanishing or explosion in recurrent neural networks and, thereby, preserve the remote memory ability of LSTM and simplify its structure. GRU can capture the dependence of words in sentences and hence is widely used in text classification, machine translation, and other tasks. GRU mainly includes two types of gates: the update gate and the reset gate. The update gate replaces the forget gate and the input gate in LSTM and the reset gate stores the information that may be forgotten easily.

### 3.2. Model structure

**Figure 2:**
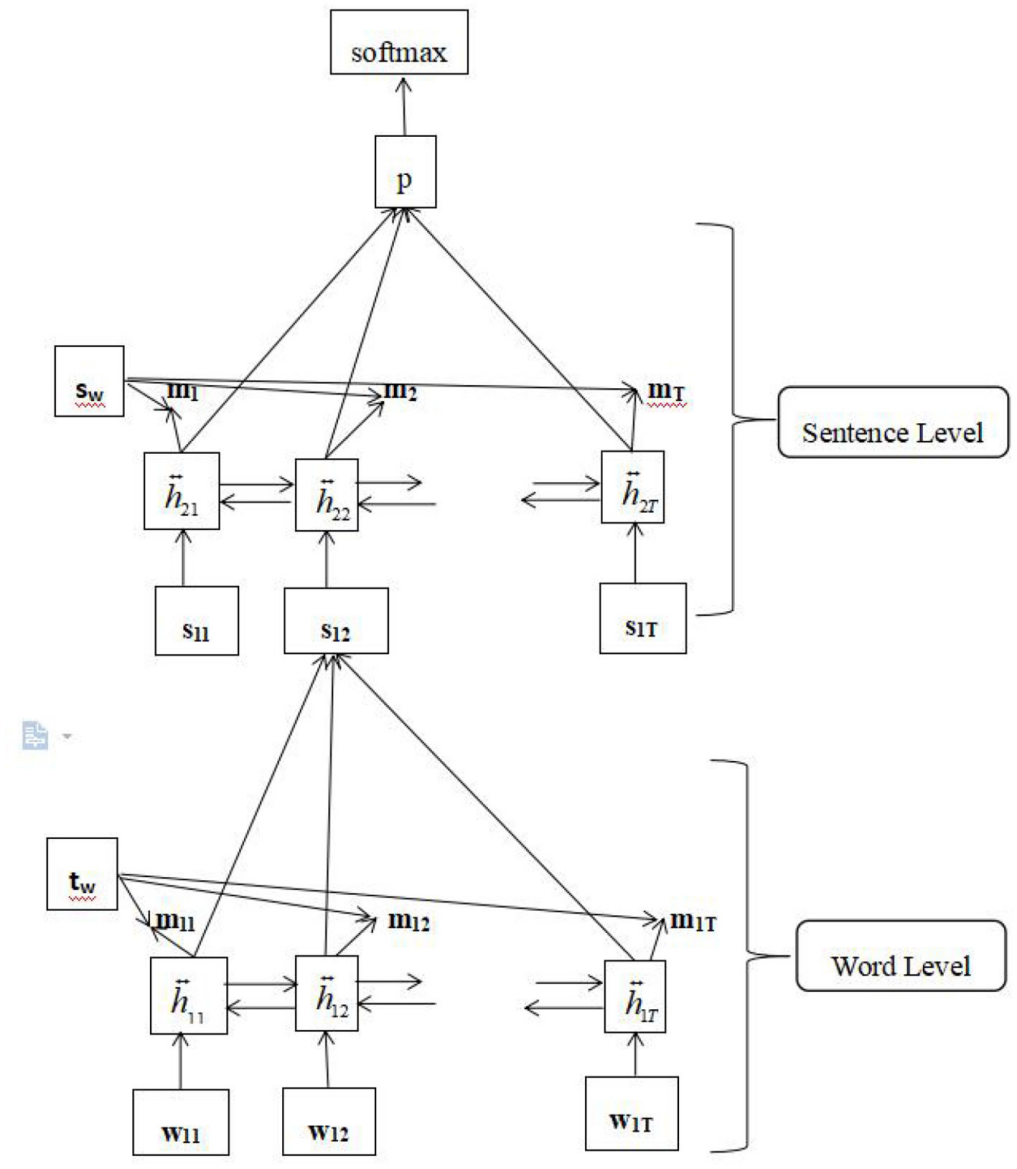
The model architecture of the attention network.

The attention mechanism (Vaswani et al., 2017) can select the most valuable information from texts. In the field of automatic language processing, such as machine translation and text classification, it can not only improve the performance of the model but also visualize the internal valuable information of the text. For text classification, the attention mechanism highlights the importance of words and sentences in the final classification. The entire model structure includes four parts: a word encoder, word attention, sentence encoder, and sentence attention. The structure of the model is illustrated in Figure 2.

#### 3.2.1. Word encoder

We embedded words into vectors through an embedding matrix *We*, which is used to obtain the annotation by summarizing information from two directions for words; therefore, it can incorporate contextual contents. Bidirectional GRU can obtain information representation of whole sentences from two directions.

Suppose there are *L* sentences in document *s*_*i*_, like [s_1_,s_2_,...,s_L_], the input of the model is the words in the joint set of all the sentences s_i_ with i ∈ [1, L] in the transcripts. Every sentence includes *T*_*i*_words; *w*_*it*_ is the *tth* word in the *ith* sentence. The word was mapped into vector *x*_*it*_ through an embedding matrix, *We* [Eq.(1)]. The implicit vector *h*_*it*_ was obtained by calculating the bidirectional GRU [Eq.(2)]. Full-text information can be fully obtained through a bidirectional calculation.


(1) $$\begin{array}{l} x_{it}=Wew_{it},\;t\in \left[1,T\right] \end{array}$$

(2) $$\begin{array}{l} \vec{h_{it}}=\vec{GRU}\left(x_{it}\right),\;t\in \left[1,T\right] \end{array}$$

(3) $$\begin{array}{l} \vec{h_{it}}=\overset{⃖}{GRU}\left(x_{it}\right),\;t\in \left[1,T\right] \end{array}$$

$h_{it}=\left[{\vec{h}}_{it},\vec{h_{it}}\right]$ is the final word vector that summarizes the information of the entire sentence centered on *w*_*it*_. The input is the words in the joint set of all sentences s_i_ with *i* ∈ [*1, L*] in the transcript, like [*s*_1_*, s*_2_*,…, s*_*L*_].

#### 3.2.2. Word attention

Not all words contribute equally to the representation of a sentence. Thus, we introduce an attention mechanism to extract informative words that are important to the meaning of a sentence and integrate them into the representation of sentence vectors.


(4) $$\begin{array}{l} s_{it}=\tanh\left(W_{w}h_{it}+b_{w}\right) \end{array}$$

(5) $$\begin{array}{l} m_{it}=soft\max\left({s_{it}}^{T}t_{w}\right) \end{array}$$

(6) $$\begin{array}{l} p_{i}=\underset{t}{\sum }m_{it}h_{it} \end{array}$$

where *t*_*w*_ is a high-level representation of the sentence vector and can be learned iteratively; it is initialized randomly and learned jointly during the training process. The hidden layer vector was further represented by a multilayer perceptron, that is, we obtained the representation of *s*_*it*_ as a hidden representation of *h*_*it*_. The importance of words was measured by calculating the similarity between *s*_*it*_ and the context word vector *t*_*w*_ and then standardizing it using the softmax function to obtain a normalized weight matrix *m*_*it*_; that is, we calculated the importance of the word vector *s*_*it*_ and obtained the important weight *m*_*it*_through the softmax function. Finally, we calculated the sentence vector representation *p*_*i*_as the weighted sum of words.

#### 3.2.3. Sentence encoder

Similarly, we used bidirectional GRU to encode the sentence vector *s*_*i*_.


(7) $$\begin{array}{l} {\vec{h}}_{i}=\vec{GRU}\left(s_{i}\right),\;i\in \left[1,L\right] \end{array}$$

(8) $$\begin{array}{l} {\vec{h}}_{i}=\overset{⃖}{GRU}\left(s_{i}\right),\;i\in \left[L,1\right] \end{array}$$

where *h*_*i*_ focuses on sentence *s*_*i*_ and summarizes neighboring sentences around sentence *i*, $h_{i}=\left[{\vec{h}}_{i},\vec{h_{i}}\right]$.

#### 3.2.4. Sentence attention

To highlight the contribution of important sentences to the representation of a document, the importance of sentences can be measured using the attention mechanism and the sentence-level context vector *s*_*w*_.


(9) $$\begin{array}{l} s_{i}=\tanh\left(W_{w}h_{i}+b_{w}\right) \end{array}$$

(10) $$\begin{array}{l} m_{i}=soft\max\left(\;{s_{i}}^{T}s_{w}\right) \end{array}$$

(11) $$\begin{array}{l} p=\underset{t}{\sum }m_{i}h_{i} \end{array}$$

where *p* is a document vector that summarizes the information of the sentences in a document. The process of sentence attention is initialized randomly and learned jointly during the entire training process.

#### 3.2.5. Document classification

The document vector *p* is a high-level representation of the document and can be used as a feature for text classification.


(12) $$\begin{array}{l} t=soft\max\left(wp+b\right) \end{array}$$

The loss function in this study is a negative log-likelihood of correct labels.


(13) $$\begin{array}{l} Loss=-\underset{d}{\sum }\log\;t_{dj} \end{array}$$

where j is the label of document d. Finally, the output of the model is a binary classification result obtained by using the softmax function.

## 4. Experiments

### 4.1. Pitt corpus

**Figure 3:**
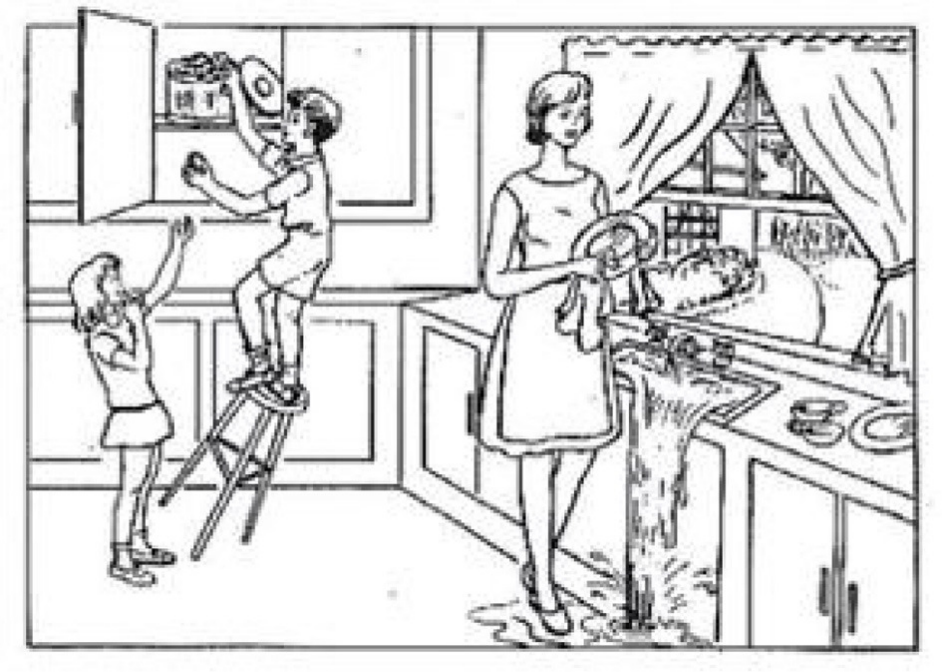
Cookie theft picture.

We performed experiments on the public Pitt Corpus of the DementiaBank (https://sla.talkbank.org/TBB/dementia/English/Pitt) (Becker et al., 1994), which was gathered longitudinally on a yearly basis. The datasets consisted of radio recordings and transcripts corresponding to the ratio of spontaneous picture description tasks produced by patients with AD and cognitively normal subjects. They were required to describe the cookie theft picture (shown in Figure 3) from the Boston Aphasia Examination (Chen et al., 2019), and the participants were all speakers of English. The transcripts of the voice recordings were gathered as part of Alzheimer's and related dementia studies by the University of Pittsburgh School of Medicine. Every audio file had an associated transcript, allowing for acoustic and lexical analyses in parallel; the speech sample was recorded and then manually transcribed at the word level using codes for the human analysis of transcripts (CHAT) coding system (MacWhinney, 2021). Every transcript came with morphosyntactic analysis automatically, such as repetition markers, description of tense, and standard part-of-speech tagging. Note that we removed utterances that had accompanying dysfluency annotations, morphological analysis, POS tags, and other associated information, leaving only pure text contents; as the deep learning model does not need to extract features manually, we aimed to create a fully automated system that does not need the participation of human annotators. After data preprocessing, 498 participants were enrolled in this study, including 242 normal controls and 256 people with possible and probable AD, and their corresponding transcripts were obtained. We divided the datasets into training sets, validation sets, and testing sets in a ratio of approximately 8:1:1. Therefore, the final number of the three datasets was 400, 50, and 48, respectively. Demographic information is shown in Table 2.

**Table 2:** Demographics of Pitt datasets.

	**CTRL (242)**	**Possible/probable AD (256)**
Age (years)	65.2 (7.8)	71.8 (8.5)
Education (years)	14.1 (2.4)	12.5 (2.9)
Gender (male/female)	86/156	90/166
Mini-mental state exam	29.1 (1.1)	18.5 (5.1)

### 4.2. Model configuration and structure

Documents were split into sentences, and every sentence was tokenized using StanfoCoreNLP (Manning et al., 2014). For word embedding, three methods were used to obtain the best performance in this study, i.e., word2vec from Google (Mikolov et al., 2013), Glove (https://nlp.stanford.edu/projects/glove/) including four word2vec files (50d, 100d, 200d, and 300d) from Stanford University, and FastText (https://fasttext.cc/docs/en/crawl-vectors.html) from Facebook. Glove and Fasttext needed a shorter training time, while word2vec required a longer time. Finally, the word embeddings were pretrained on Stanford's publicly available 100-dimensional Glove for better performance after comparison. We obtained the word embeddings on the training and validation splits and then used them to initialize *We*. The number of GRU units was set to 100 and the dense layer dimension at the word level was set to 50. The proposed model was trained on a fixed 10 epochs and evaluated on the validation sets at every epoch. Word weight and context weight were initialized randomly according to a normal distribution (mean = 0, std = 0.1). Similarly, sentence weight and context weight were also initialized randomly according to a normal distribution with mean and std being 0 and 0.1, respectively. Word bias and sentence bias were initialized randomly in the training stage. We applied an Adam optimizer with a 0.01 learning rate; the dropout to the output of all the functional layers was used, and the dropout rate was set to 0.35 for all the layers. All the aforementioned parameters were trained on the training sets and the best model was selected based on the accuracy of the validation sets. All the aforementioned parameters can be applied to the other models.

### 4.3. Results and analysis

In this study, we evaluated the effectiveness of our model with a five-fold cross-validation. That is, four sets were used as training sets and one as the test set, the results of which were summarized, and the average value was calculated. The relationship between the actual and predicted classes is presented in Table 3 and the metric formulas of accuracy, precision, recall rate, and F1 score are shown in Eq. (18)–(21).


(14) $$\begin{array}{c} Accuracy=\frac{TN+TP}{TN+FP+FN+TP} \end{array}$$

(15) $$\begin{array}{c} Precision=\frac{TP}{TP+FP} \end{array}$$

(16) $$\begin{array}{c} Recall=\frac{TP}{TP+FN} \end{array}$$

(17) $$\begin{array}{c} F1=\frac{2TP}{2TP+FP+FN} \end{array}$$

**Figure 4:**
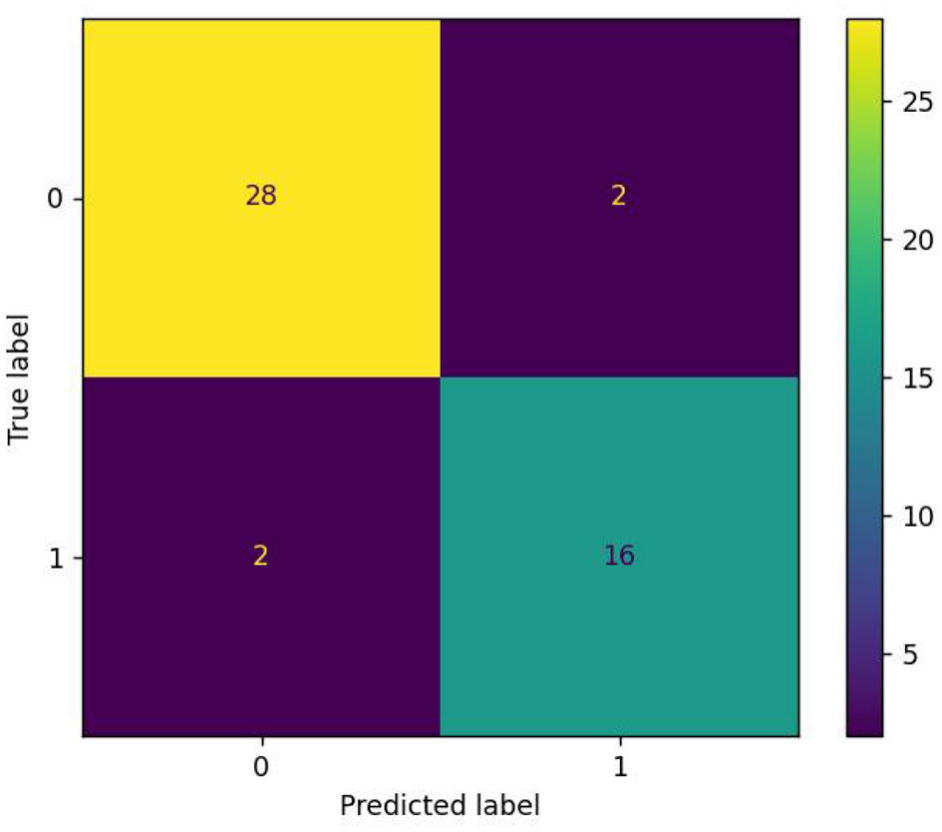
Result of the confusion matrix.

Table 4 shows the performance of the studies with Pitt datasets in this area. Of course, these datasets may include different subsets of the Pitt Cookie Theft corpus, and the results summarized in Table 4 are not always comparable. In addition, these articles are not exhaustive because of our limited ability. Of all the studies in Table 4, the first set of studies (Becker et al., 1994; Clarke et al., 2013; Yancheva and Rudzicz, 2016; Sirts et al., 2017; Hernández-Domínguez et al., 2018; Fraser et al., 2019; Li et al., 2019; Antonsson et al., 2021; R'mani and James, 2021; Zehra et al., 2021) used a feature extraction + machine learning method, and the best accuracy was 85.4%. The second set of studies (Karlekar et al., 2018; Orimaye et al., 2018; Fritsch et al., 2019; Pan et al., 2019; Balagopalan et al., 2021; Guo et al., 2021; Meghanani et al., 2021) used deep learning methods, of which the best accuracy was 91.1% (Karlekar et al., 2018). The rest of the studies (Yuan et al., 2020; Pranav and Veeky, 2021; Roshanzamir et al., 2021; Tristan and Saturnino Analysis, 2021) used deep learning models in combination with acoustic features or linguistic features. The study by Yuan et al. (2020) obtained the best accuracy of 89.6%, the highest in Interspeech 2020. Our method obtained the best accuracy of 91.6%, which is 0.5% higher than the best performance of the study by Karlekar et al. (2018). The image of the confusion matrix of our study is shown in Figure 4, and only two AD and two NC in 48 testing sets were not recognized correctly.

**Table 3:** Relationship between the predicted and true classes.

	**True class**	
Predicted class	Positive	Negative
Positive	True positive (TP)	False positive (FP)
Negative	False negative (FN)	True negative (TN)

**Table 4:** AD vs. CTRL classification scores(%) on Pitt datasets.

**Method**	**Embedding**	**Classifier**	**Precision**	**Recall**	**Accuracy**	**AUC**	**F1**
Antonsson et al. (2021)	Semantic features	SVM	-	-	-	0.93	-
Clarke et al. (2013)	286 Linguistic features	-	-	-	50–78 for MCI vs. HC, 59–90 for AD vs. HC, and 62–78 for AD+MCI vs. HC	-	-
R'mani and James (2021)	x-vectors and i-vectors features (Roark et al., 2011)	Random Forests and SVM	-	-	85.4	-	-
Zehra et al. (2021)	Hand-Craft acoustic and linguistic features	Logistic Regression	-	-	85.4	-	-
Becker et al. (1994)	35Hand-Crafted Feature	Logistic Regression (LR)	-	-	81.92	-	-
Yancheva and Rudzicz (2016)	12Cluster-Based Features+LS&A	Random Forest	80.00	80.00	80.00	-	80.00
Sirts et al. (2017)	Cluster+PID+SID Features	LR	74.4 ± 1.5	72.5 ± 1.2	-	-	72.7 ± 1.2
Hernández-Domínguez et al. (2018)	105Hand-Crafted Features	SVM	81.00	81.00	79.00	-	81.00
Li et al. (2019)	185Hand-Craft Features	LR	-	-	77	-	-
Fraser et al. (2016)	Info and LM Features	SVM	-	-	75	-	77
Fritsch et al. (2019)	n-gram	NNLM+LSTM	-	-	85.6	-	-
Balagopalan et al. (2021)	-	BERT	-	-	83.33	-	-
Guo et al. (2021)	-	BERT	-	-	82.1	-	-
Meghanani et al. (2021)	-	FastText	-	-	83.3	-	-
Karlekar et al. (2018)	POS-tagged data	CNN-RNN	-	-	91.1	-	-
Orimaye et al. (2018)	n-grams	D2NN	-	-	88.9	-	-
Pan et al. (2019)	GloVe Word Embedding Sequence	BiLSTM|GRU Hierarchical Attention	84.02	84.97	-	-	84.43
Yuan et al. (2020)	Encoding of pauses+ERNIE Embedding	ERNIE	-	-	89.6	-	-
Tristan and Saturnino Analysis (2021)	Word cooccurrence graphs	Machine Learning	-	-	66.7	-	-
Roshanzamir et al. (2021)	BERT_Base_	LR	90.31 ±7.36	76.52 ±8.06	84.46 ±6.31	-	82.72 ±7.21
Roshanzamir et al. (2021)	Bert_Large_	LR	90.57 ± 3.18	84.34 ± 7.58	88.08 ± 4.48	-	87.23 ± 5.20
Pranav and Veeky (2021)	Linguistic features	Deep learning	-	-	-	88	-
Our method	GRU	Softmax	-	-	**91.6**	-	-

### 4.4. Ablation study on attention network

We validated the effectiveness of every part by ablation study, as illustrated in Table 5. First, removing the word level (-Word) leads to a 1.4% performance drop for Pitt datasets. Similarly, removing the sentence level (-Sentence) leads to a 2.3% performance drop, which is more significant than removing the word level. From the ablation experiment, we can demonstrate that the word level and sentence level are essential to our model.

**Table 5:** Ablation study on our model.

**Method**	**Accuracy**	**Drop**
Our Model	91.6	-
(-Word)	90.2	1.4
(-Sentence)	89.3	2.3

### 4.5. Visualization of attention features

**Figure 5:**
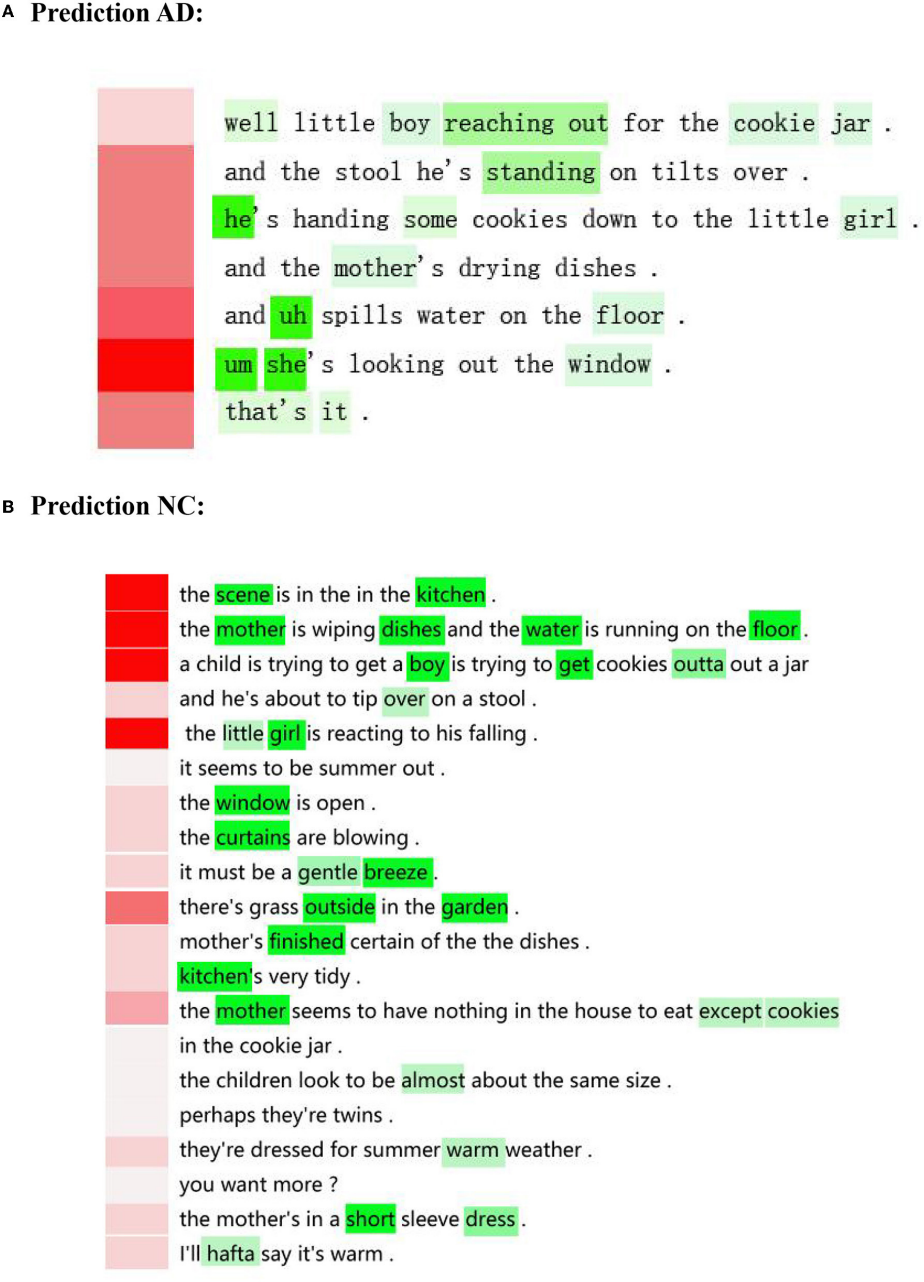
An example of AD and NC from Pitt dataset. **(A)** Prediction AD. **(B)** Prediction NC.

We normalized the word weight by sentence weight to make sure that only important words in important sentences are emphasized because of the hierarchical structure. To validate that our proposed model can select formative words and sentences, we visualized the contextual attention features shown in Figure 5. Each line is a sentence; green denotes the word weight and red denotes the sentence weight. The study by Liu and Yuan (2022) indicates that a general and integral expression for normal should include the following seed words: boy, girl, woman, cookie, stool, sink, overflow, fall, window, curtain, plate, cloth, jar, water, cupboard, dish, kitchen, garden, take, wash, reach, attention, and see. In the AD group, we found three problems in linguistic expression. For the first one, our model only referred to a few seed words such as “boy”, “girl”, “mother”, “floor”, and “window”, and the description was much shorter compared to that of the NC group. The participant cannot describe the picture completely which affects the adequacy of discourse information to some extent. For the second one, our model localized the key colloquial words such as “uh”, and “um”; the study by Yuan et al. (2020) indicates that people with AD use more “uh” and “um” than NC. There is usually a pause after “uh” and “um” and the participant may not find appropriate words or sentences to express himself, which finally influences verbal fluency. For the third one, our model accurately localized personal pronouns such as “he” and “she”, as well as the corresponding sentences, which means that people with AD may have a word-finding difficulty and can only use he or she to replace, which finally influences the sentence expression and meaningful output.

In the normal group, our model selected more seed words, such as scene, kitchen, mother, dish, water, garden, boy, girl, mother, window, curtain, breeze, water, and their corresponding sentences, indicating a rich vocabulary and integrated semantic expression. In addition, some attributive words that our model selected include “little”, “short”, “gentle”, and “almost”, manifesting a sufficiency of discourse information and the coherence of discourse.

## 5. Conclusion

Many studies on AD diagnosis using language focused on the deep learning method (Liu et al., 2021, 2022; Chen and Liu, 2022) as the traditional feature extraction method is blind, lacks integrity, and has a relatively worse performance compared with the deep learning method. Meanwhile, with the development of deep learning, new methods such as contrast learning, unsupervised learning, and multimodal feature fusion can be used to differentiate AD from normal controls.

This study used the deep learning method combined with the attention mechanism to identify important words in a sentence to form sentence representation and important sentences in a document, which formed the representation of the whole document. We combined contextual features with the attention mechanism and studied the classification of implicit effective sentences based on the bi-GRU model and attention mechanism. Of course, the encoder of bi-GRU in our model can be replaced by other models, such as RNN and LSTM. Owing to the difference in expression between implicit and explicit texts, the proposed model can learn fuzzy implicit sentiment with contextual attention features to improve classification performance. Compared with the general classification model, our model can extract more valuable information based on word and sentence levels. Experimental results on public Pitt datasets show the superiority of our model to other classification models in AD diagnosis. Meanwhile, deep learning models are considered “a blind box” (Meghanani et al., 2021), the interpretability of which is not better than that of the machine learning method as we cannot obtain the feature information that humans can understand from these models. However, our work can be visualized further as we may select more informative words and sentences that affect the classification effect, which may provide some references for the detection and rehabilitation of cognitive dysfunction sufferings from the perspective of linguistics.

However, our model may ignore some potential risks. For example, the corpus we used may contain recordings taken over multiple visits from the same patient, which might bias the model because the training sets and testing sets may be from the same patient. To eliminate this bias, the studies (Luz et al., 2020, 2021), for example, employed the one-to-one matching approach and propensity score matching strategy, respectively. The datasets of the ADReSS challenge in 2010 were created precisely for avoiding this and other potential sources of bias (such as gender and age). In our future study, we will take effective measures to eliminate these potential biases.

## Data availability statement

The original contributions presented in the study are included in the article/supplementary material, further inquiries can be directed to the corresponding author/s.

## Ethics statement

Written informed consent was obtained from the individual(s) for the publication of any potentially identifiable images or data included in this article.

## Author contributions

ZY gave some good suggestions and revised the parameters of the model. YC revised the background introduction. All authors contributed to the article and approved the submitted version.
